# Metagenomic Analysis of Cyanobacteria in an Oligotrophic Tropical Estuary, South Atlantic

**DOI:** 10.3389/fmicb.2018.01393

**Published:** 2018-06-26

**Authors:** Helen M. de Jesus Affe, Janaina Rigonato, José M. de Castro Nunes, Mariângela Menezes

**Affiliations:** ^1^Laboratório de Algas Marinhas, Instituto de Biologia, Universidade Federal da Bahia, Salvador, Brazil; ^2^Centro de Energia Nuclear e Agricultura, Universidade de São Paulo, Piracicaba, Brazil; ^3^CEA, Centre de Sequençage Genoscope, Institut de Biologie François Jacob, Evry, France; ^4^Laboratório de Ficologia, Departamento de Botânica, Museu Nacional, Universidade Federal do Rio de Janeiro, Rio de Janeiro, Brazil

**Keywords:** marine cyanobacteria, metagenomic diversity, functional profile, shotgun, Camamu Bay

## Abstract

This study assessed the species composition, distribution, and functional profiles of cyanobacteria in Camamu Bay, a tropical oligotrophic estuarine system on the northeast coast of Brazil, using shotgun metagenomic sequencing. Surface-water samples were evaluated in two different rainfall periods (rainy and dry seasons), at nine stations in the three hydrodynamic regions of the bay. At a fixed sampling station, on each season, samples were taken over a tidal cycle at 3-h intervals over 12 h. A total of 219 cyanobacterial taxa were identified, demonstrating a diverse community of freshwater, euryhaline, and marine cyanobacteria. The genera of greater relative abundance, *Synechococcus* and *Prochlorococcus*, corresponded to the picoplankton fraction. Although Camamu Bay has conspicuous marine characteristics, the contribution of freshwater during the rainy season caused variation in cyanobacteria community, with an increase in species richness. Due the high prevalence of *Synechococcus* (90% of the sequences), the functional analysis revealed only minor differences in gene content between the dry and rainy seasons. In both rainy and dry seasons, an increase in *Prochlorococcus* relative abundance occurred during high tide, demonstrating the tidal influence in the bay. The environmental characteristics of the bay provide niche conditions for a wide variety of cyanobacteria, including freshwater, euryhaline, and marine strains.

## Introduction

Estuaries are aquatic coastal ecosystems connecting rivers to the ocean. These systems receive large inputs of nutrients and are strongly influenced by tides, where the ocean water is diluted by freshwater from continental drainage (Pritchard, 1967). Estuarine systems vary widely in their physical and chemical properties, altering habitats, and community composition, and therefore biogeochemical processes (Ghosal et al., 2000; Attrill and Rundle, 2002; Brunet and Lizon, 2003; Cloern et al., 2016). Phytoplankton is the major source of carbon in estuaries, in addition to the high input of organic compounds carried primarily by continental freshwaters (Cloern et al., 2014). They play a fundamental role in the processes and biogeochemical cycles that maintain the functioning of these systems (Cloern and Dufford, 2005; Cloern et al., 2014).

Cyanobacteria constitute a dominant prokaryotic fraction of the marine phytoplankton community (Waterbury et al., 1979; Fogg, 1982; Capone et al., 1997; Paerl, 2012; Shih et al., 2013; Coutinho et al., 2016). They contribute to global primary production and regulate the biogeochemical cycles of several elements, including nitrogen through biological nitrogen fixation (Falkowski and Woodhead, 1992; Falkowski, 1997; Whitehead et al., 2014). Several studies have reported shifts in the cyanobacterial community, particularly picocyanobacteria, based on analysis of the V3–V4 region of the 16S rRNA gene, mainly using the primers CYA359F (forward), CYA781R (a), and CYA781R (b) (reverse) (Nübel et al., 1997). For example, studies based on partial 16S rRNA sequences suggest high geographical endemism and rapid diversification of *Synechococcus* populations in the Baltic Sea (Haverkamp et al., 2009).

Advances in sequencing technologies and analytical tools to quantify differences among cyanobacterial communities have supported an increase in the number of microbial-diversity studies. They have also improved knowledge of the role of these organisms in the functioning and maintenance of marine ecosystems (Turnbaugh et al., 2007; Raes and Bork, 2008; Logares et al., 2009; Caporaso et al., 2012; González et al., 2012; Grosskopf and Soyer, 2014; Ininbergs et al., 2015). In particular, metagenomic analyses of environmental samples have increased the accuracy of quantitative approaches to cyanobacterial communities, allowing evaluations of their taxonomic composition, the relative abundance of their genes, and their metabolic profiles (Venter et al., 2004; DeLong et al., 2006; Sunagawa et al., 2015). These analyses have also improved the understanding of non-culturable species and their association with a variety of environmental parameters in different water bodies (Galand et al., 2009) and depths (Johnson et al., 2006; Brown et al., 2009; Sharpton, 2014; Díez et al., 2016).

Studies on the diversity and structure of marine cyanobacterial communities using metagenomic approaches have demonstrated the overall dominance of picocyanobacteria belonging to the genera *Synechococcus* and *Prochlorococcus* (Partensky et al., 1999; Garcia-Pichel et al., 2003; Flombaum et al., 2013; Díez et al., 2016). Cyanobacterial communities in estuarine and other brackish environments remain poorly studied through molecular techniques, especially those involving high-throughput sequencing (Balzano et al., 2015; Ininbergs et al., 2015; Celepli et al., 2017). Metagenomic studies in these environments may reveal important aspects of the genetic and functional biodiversity of the microbial communities, considering the variability resulting from the mixing of marine and freshwater (Muylaert et al., 2009; Cloern et al., 2016).

The Brazilian coastline contains over a hundred estuaries, from the equator in the north to the temperate south (Bernardino et al., 2016). Studies applying metagenomic techniques to assess the diversity of cyanobacteria in these systems are incipient. The few previous studies were restricted to an analysis of bacterial diversity, for instance in Guanabara Bay, a hypereutrophic estuary in southeastern Brazil (Gregoracci et al., 2012).

Here, we present the first metagenomic shotgun approach performed in a Brazilian tropical oligotrophic estuarine system. Camamu Bay is a system formed by three hydrodynamic regions, delimited by the drainage area of five tributary streams, besides the main channel, where all exchanges between the bay and the adjacent coastal area occur (Menezes, 2011; Amorim et al., 2015). We hypothesized that the cyanobacteria community would show spatial and temporal changes in response to the influence of rivers on the estuary hydrodynamic regions and the force of the tides at the entrance of the system. The objective of this study was to characterize the diversity of cyanobacteria in this tropical estuarine system over two different rainfall periods (rainy and dry seasons) and through complete tidal cycles. Camamu Bay is a relatively pristine system, and the expansion of knowledge of its biodiversity will lead to a better understanding of the community patterns of cyanobacteria in the natural environment.

## Materials and methods

### Study area

**Figure 1:**
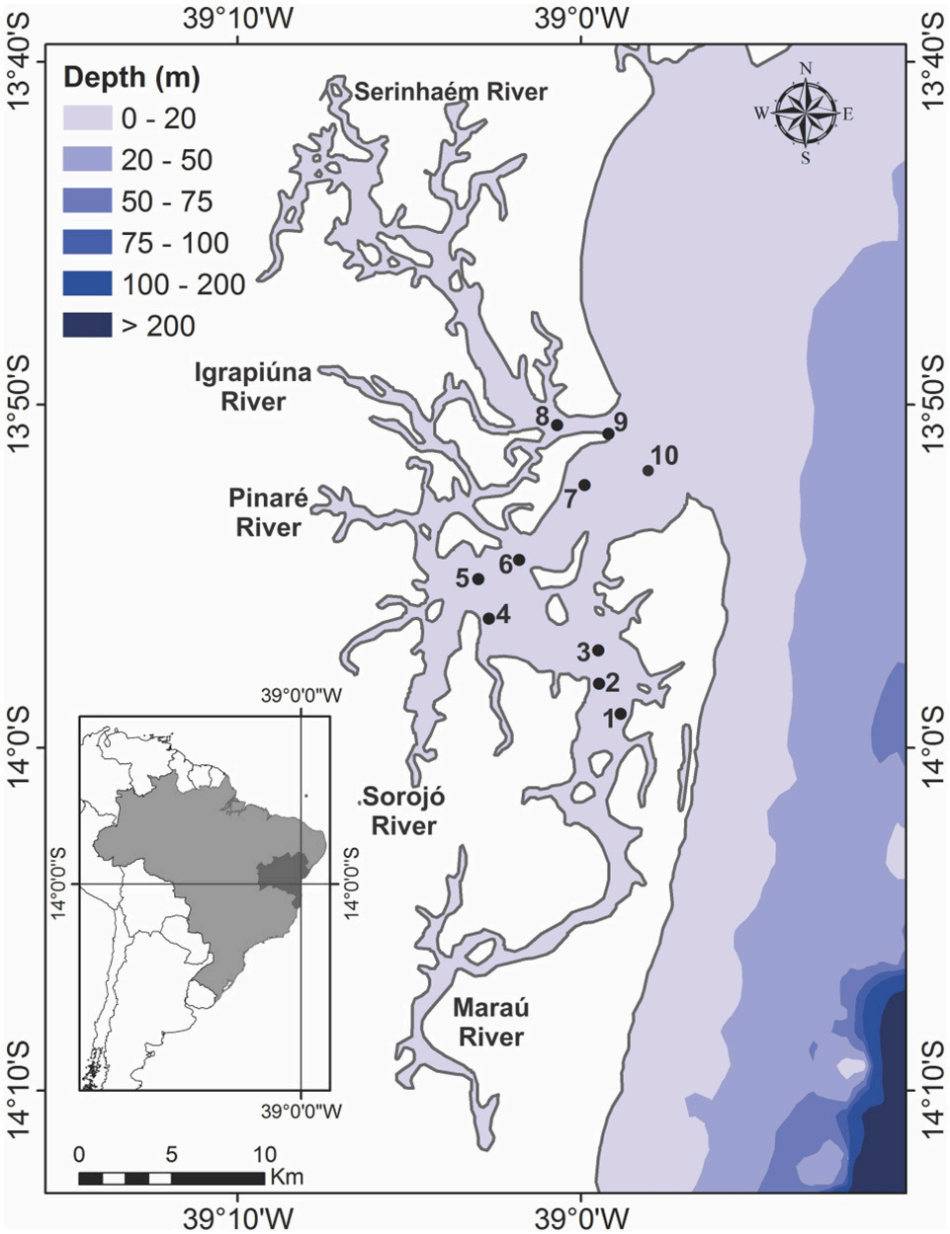
Map showing the 10 sampling locations in Camamu Bay: spatial distribution in the hydrodynamic regions point 1–3 (MAR), 4–6 (CEN), 7–9 (SER), and the fixed sampling station (FS), point 10 (sampling over a tidal cycle).

Camamu Bay is situated on the central coast of the state of Bahia, Brazil (Figure 1), a region with a warm and humid climate, 25°C annual mean temperature, and high rainfall, between 2,400 and 2,600 mm yr^−1^ (Centro de Recursos Ambientais and Assessoria de Comunicação Social, 2007). This estuary is shallow (mean depth 5 m), with well-mixed water, especially during spring tides (Amorim et al., 2011, 2015).

The hydrodynamic circulation inside the bay is forced by tides, with a maximum range of 2.7 m and current velocities from 0.6 to 1.2 m s^−1^ (Amorim et al., 2015). The principal rivers entering the bay are the Serinhaém River (SER) in the northern part, a shallow channel with 7.3 m mean depth; the Igrapiúna, Pinaré, and Sorojó rivers in the central region (CEN), with 3 m mean depth and maximum depth of 7 m in the river channels; and the Maraú River (MAR) in the southern part, with 6.2 m mean depth (Oliveira et al., 2002; Hatje et al., 2008; Amorim et al., 2011). The Maraú channel is a partially mixed system; the Serinhaém channel is well mixed during spring tides and somewhat mixed during neap tides, and the water is well mixed from the bay entrance to mid-bay. The system has a seasonally controlled cleaning/purifying capacity; the water is renewed every 90 days in the dry season and every 30 days in the rainy season (Amorim et al., 2015).

### Sampling and analyses

Sampling was conducted during spring tides in two different rainfall periods. The first period was in October 2014, after cumulative precipitation of 198.2 mm (rainy season), and the second was in January 2015, after cumulative rainfall of 93.2 mm (dry season), for the 30-day period before each sampling. Rainfall data were obtained from the National Meteorological Institute (INMET)^1^, based on records of the automatic meteorological station (13°54′S, 38°58′W) in the municipality of Maraú, Bahia. The discharges of the principal tributary streams were calculated using the equation of Smith et al. (1999).

To evaluate possible influences of the tributary streams on the bay system, three sites were sampled in each of the hydrodynamic regions (SER, CEN, and MAR) of the bay, with three points sampled at each site (Figure 1, points 1–9). To determine the influence of the tide on the cyanobacterial community, time-series samples were taken at a fixed sampling station (FS), at intervals of 3 h, for a total of 12 h over two tidal cycles. The fixed sampling station, at the bay entrance (Figure 1, point 10), is representative of the entire water exchange of the system, following hydrodynamic modeling of the area according to Menezes (2011). At each sampling station and each hour of the tidal cycle, the temperature, salinity, and dissolved oxygen were measured *in situ*, using a multiparameter meter (Hanna HI 9829, São Paulo, SP, Brazil). Water transparency was estimated using a Secchi disc.

For nutrient analyses, water samples (5 L) were collected at the subsurface, using a Van Dorn bottle. Samples were stored in polyethylene flasks, pre-washed (HCl and distilled water), and immediately filtered using a vacuum pump (PRÓ-TOOLS 2110, Porto Alegre, RS, Brazil) with fiberglass filters (Whatman GF/F, 0.7 μm pore, Sigma-Aldrich, St. Louis, MO, USA). Aliquots of 250 mL of the filtered volume of each sample were kept frozen (−20°C) for at most 2 weeks until the analyses were conducted. The dissolved inorganic nutrients (nitrite, nitrate, ammonium, phosphate) were analyzed (24 samples/period) by the spectrophotometric method, according to Grasshoff et al. (1983).

For DNA extraction, water samples (1 L) were collected at the subsurface, using a Van Dorn bottle, and immediately filtered through a polycarbonate membrane (Merck GS, 0.22 μm pore, Darmstadt, Germany) with a vacuum pump. The filters were stored in microtubes kept on ice during transport to the laboratory, where they were stored in an ultra-freezer at −80°C (Thermo Scientific, Waltham, MA, USA) until DNA extraction.

### Metagenomic library preparation, sequencing, and bioinformatic analyses

Genomic DNA was extracted by cutting the filter into small pieces with sterilized scissors and then transferring the fragments to tubes containing beads, following the manufacturer's instructions in the PowerSoil DNA Isolation Kit (MoBio Laboratories, Carlsbad, CA, USA).

The metagenomic libraries were prepared by fragmentation of total DNA using a Nextera XT DNA Sample Preparation Kit (Illumina, Inc., San Diego, CA, USA), using 1 ng of total DNA of each sample. After fragmentation, the DNA was labeled with adapters using the Nextera XT Index Kit (Illumina), and purified with the Agencourt AMPure XP Beads Kit (Beckman Coulter, Inc., Brea, CA, USA). The entire preparation procedure followed the Nextera DNA Sample Preparation Guide (Illumina).

Quantification of the DNA libraries was performed by real-time PCR, using the KAPA Library Quantification Kit for Illumina (KAPA Biosystems, Wilmington, DE, USA), according to the manufacturer's protocol. The library obtained from each sample was normalized to obtain equimolar amounts of DNA and later grouped in a single pool. The libraries were sequenced on the MiSeq platform (Illumina) with the 600-cycle MiSeq reagent kit v3 (Illumina), at the Center for Functional Genomics Applied to Agriculture and Agroenergy at the University of São Paulo, ESALQ, Piracicaba.

After sequencing, the paired-end reads were merged using the Flash tool (Fast Length Adjustment of Short Reads) version 1.2.7 (http://ccb.jhu.edu/software/FLASH/). The sequences were filtered by quality (Phred 20) and size (minimum length 80 bp) using Seqyclean 1.3.12 (http://cores.ibest.uidaho.edu/software/seqyclean).

Metagenomic data were analyzed using MEGAN6 (Huson et al., 2016). Sequences were aligned against the NCBI-nr database (as of August 2017) using DIAMOND (Buchfink et al., 2015) and the taxonomic assignation of the reads was performed based on the lowest common ancestor (LCA) algorithm (Huson et al., 2016). In this approach, reads that aligned with conserved sequences were assigned to high-level taxa (for instance Phylum), whereas sequences aligned to species-specific sequences were assigned to lower taxa (for instance genus/species) (Huson et al., 2016). The parameters min Score = 50, top Percent = 10, max Expected = 0.01, and Percent to cover = 100 were used as program default values as detailed by Huson et al. (2007), and all sequences assigned to cyanobacteria were extracted from the total data set. Functional classification was performed based on the SEED database (Overbeek et al., 2005).

The metagenomic reads were mapped against the *Synechococcus* sp. CB 0205 genome (NZ_ADXM00000000.1), using the bowtie2 tool (Langdon, 2015). The genome coverage was evaluated using genomecov implemented in beedtools toolset (Quinlan, 2014), considering 5x of sequencing coverage.

The sequences are available in the MG-RAST v4.0.3. metagenomic analysis server (http://www.mg-rast.org) under the unique identifiers: 4696532.3, 4696533.3, 4696534.3, 4696535.3, 4696536.3, 4696537.3, 4696538.3, 4696539.3, 4696540.3, 4696541.3, 4696542.3, 4696543.3, 4696545.3, 4696546.3, 4696547.3, 4696549.3, 4696551.3, 4696553.3, 4696555.3, 4696556.3, 4696557.3, 4696558.3, 4696559.3, 4696560.3, 4696561.3, 4696563.3, 4696564.3, 4696565.3, 4696566.3, 4696567.3, 4696568.3, 4696569.3, 4696570.3, 4696571.3, 4696572.3, 4696573.3, 4696574.3, 4696575.3, 4696576.3, 4696577.3, 4696578.3, 4696579.3, 4696580.3, 4696581.3, 4696582.3, and 4696583.3.

### Statistical analyses

The number of reads for taxonomic and functional data was normalized by relative abundance, dividing each count by the total number of assigned reads per library to prevent bias generated by different sequencing coverage. Cyanobacterial diversity was inferred based on species richness (S), Shannon diversity index (H′), and Pielou species evenness (J′). Samples were clustered combining yjr Bray-Curtis dissimilarity and Ward methods. Analysis of variance was performed with a Kruskal-Wallis test after checking the assumptions for parametric analyses (normality and homoscedasticity) using the Shapiro-Wilk and Levene tests, followed by the value multiple-comparison tests to assess the occurrence of significant differences (*p* < 0.05).

A multivariate analysis of redundancy (RDA) was used to evaluate the influence of abiotic variables on the cyanobacterial community in the bay. To quantify the relative contributions of the abiotic variables to the explanation of community composition, a variation-partitioning analysis was performed (Borcard et al., 1992; Peres-Neto et al., 2006). The environmental data were separated into four groups: (1) water flow; (2) temperature; (3) nutrients (nitrite, nitrate, and phosphate); and (4) physical and chemical variables (salinity, transparency, and dissolved oxygen). Environmental data were standardized, and the Hellinger transformation was applied (Legendre and Gallagher, 2001). All statistical analyses were carried out in the R environment, with the Vegan packages (R Core Team, 2016). The STAMP program was used for statistical analysis of functional data (Parks and Beiko, 2010; Parks et al., 2014).

Samples collected from site CEN in the dry season were discarded, due to the low quality of sequencing.

## Results

### Environmental variables

The water temperature in the rainy season was 26 ± 0.12°C on average. The great freshwater input (~13.5 m^3^ s^−1^) in this period increased the oxygen content of the estuarine water, reaching saturation (>100%), and increased the concentrations of dissolved inorganic nutrients. The concentration of nitrogen compounds (nitrate, nitrite) was 0.84 ± 0.3 μM on average, but for all samples, the ammonium concentration was below the detectable level (<0.01 μM). Phosphate concentration was 0.44 ± 0.04 μM and water-column transparency was 1.6 ± 0.2 m on average.

During the dry season, the low freshwater input (~2.6 m^3^/s) into the bay led to subsaturation of dissolved oxygen (~60%). Lower nutrient concentrations were observed for nitrogenous forms (0.67 ± 0.2 μM), but not for phosphate (0.42 ± 0.02 μM). The temperature was higher during this season (31 ± 0.3°C), and water-column transparency (2.5 ± 0.4 m) also increased compared to the rainy period. The salinity remained similar in both seasons (32 ± 1.6 on average).

### Taxonomic composition

The lowest common ancestor algorithm (LCA) was used to assign the reads taxonomically. This implies that for the subsequent analyses, only the reads assigned to infra-levels integrate the data set for the statistical analyses. A summary of the number of reads for each taxonomic level is provided in the supplementary material (Supplementary Table 1). In total, 219 species comprised the cyanobacterial community in Camamu Bay (Supplementary Table 2). The taxonomic assignments showed a large number of sequences attributed to different strains of the picocyanobacteria *Prochlorococcus* (58 strains), followed by *Synechococcus* (34 strains) (Supplementary Table 2). Sequences of *Synechococcus* belonged to three subclades: 5.1 (*Synechococcus* sp. WH 8109, *Synechococcus* sp. RS 9916, and *Synechococcus* sp. CC 9605), 5.3 (*Synechococcus* sp. RCC 307), and 5.2 (*Synechococcus* sp. CB 0205). The latter comprised 41% of the sequences that could be assigned to species level, and ranged from 12.3% of to 7.8% of the reads assigned to phylum level. About 10% of the total cyanobacterial metagenomic reads from Camamu Bay samples were mapped against the CB0205 draft genome (Supplementary Table 2). Using a cutoff of 5x sequencing depth coverage (only loci with at least 5 mapped reads) between 0.78 and 7.32% (on average 0.89%) of the CB0205 genome was covered. Using 1x sequencing coverage, on average 16.70% (3.9–40.96%) of the genome was covered.

**Figure 2:**
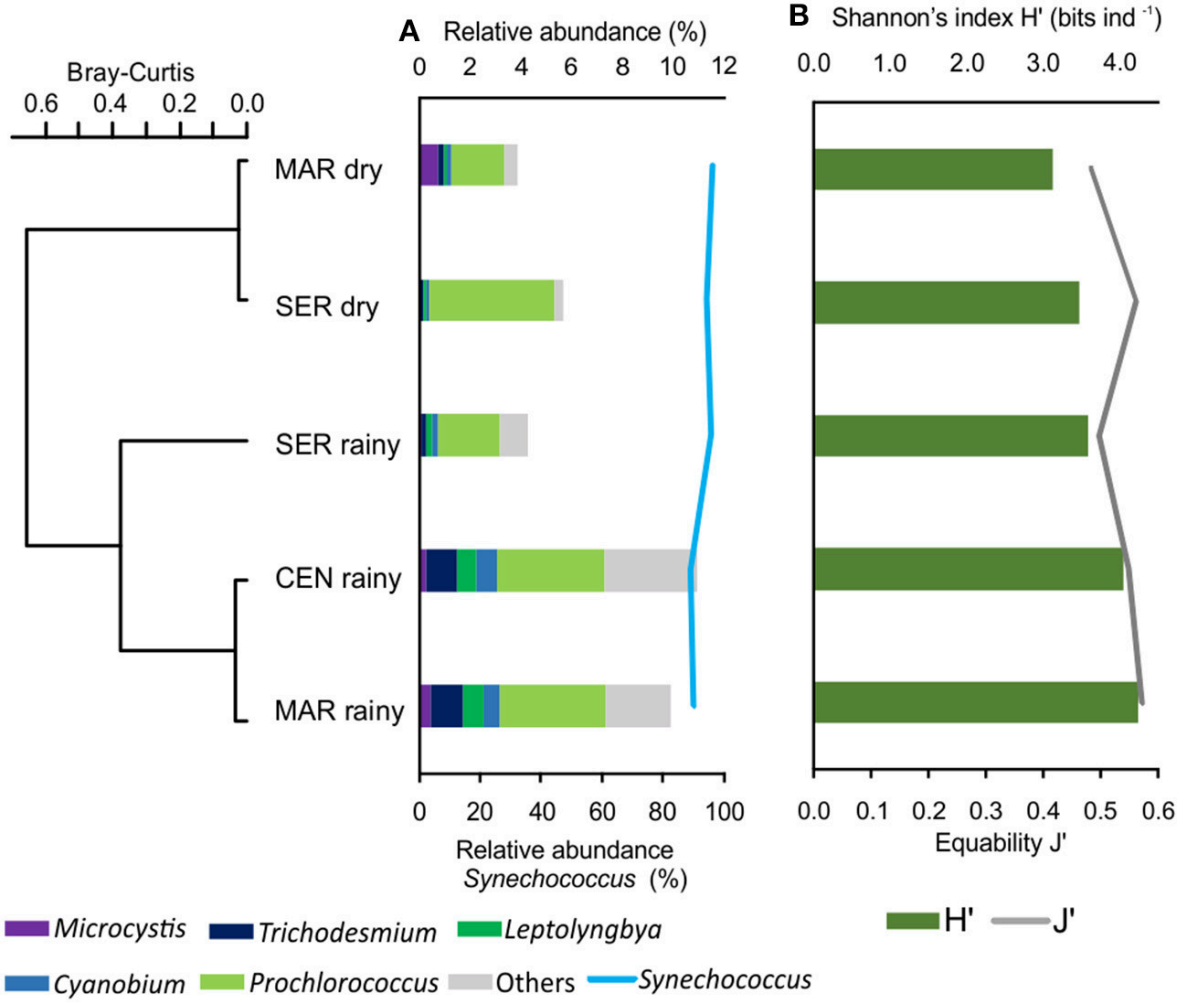
Cluster of hydrodynamic regions between rainy and dry seasons as a function of the variation of the relative abundance of cyanobacteria **(A)** and diversity (Shannon index) and Pielou evenness **(B)**.

*Synechococcus* encompassed almost 90% of the sequences assigned to genus level retrieved in all samples, followed by *Prochlorococcus* with 5% of the sequences. Besides, *Microcystis* (0.3%), *Leptolyngbya* (0.6%), *Cyanobium* (0.7%), *Trichodesmium* (0.8%), and other genera were also observed, together comprising 2% of the sequences (Figure 2A; Supplementary Table 3).

Although detected at lower levels, *Microcystis* was observed mainly at the Maraú site, in a higher proportion in the dry season and *Trichodesmium* was mostly observed in the Maraú and Central samples, and was more pronounced in the rainy season (Figure 2A).

Cyanobacterial taxonomic analysis demonstrated differences in the community composition between the dry and rainy seasons, with an increase in the number of sequences assigned to cyanobacteria in the latter (*p* = 0.0002), especially for the Central and Maraú sites. No significant differences were observed in the relative abundance of taxa (*p* = 0.43) among hydrodynamic regions (MAR, CEN, and SER) in the two seasons analyzed (Figure 2A).

The spatial distribution of cyanobacteria strains in the bay was relatively homogeneous, mainly due to the dominance of *Synechococcus* in all samples. The Shannon diversity index (H′) and evenness ranged from 3.6 to 4.2 bits ind^−1^ and 0.5 and 0.6 in the rainy season and from 3.1 to 3.4 bits ind^−1^ and 0.5 in the dry season, respectively (Figure 2B). A more pronounced variation in species richness occurred in the rainy season, with 162 (±12) taxa, while only 81 (±13) taxa were identified in the dry season (Supplementary Table 1).

**Figure 3:**
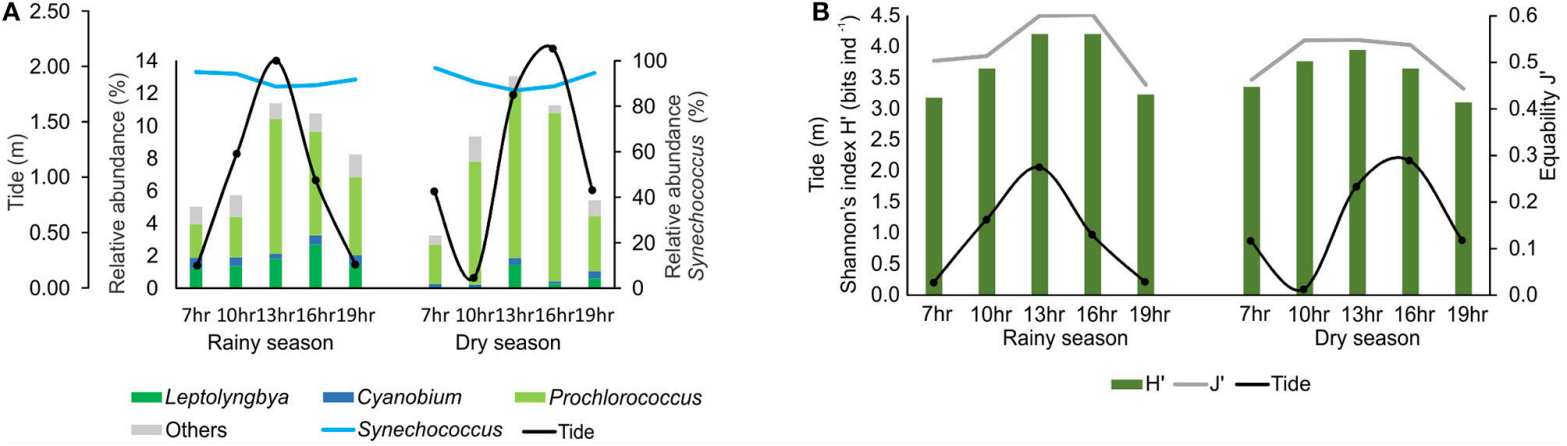
Variation of relative abundance **(A)** and diversity (Shannon index) and Pielou evenness **(B)** of cyanobacteria reads in tidal cycles.

The composition of the cyanobacteria community was similar during tidal cycles, with *Synechococcus* dominating all sampling times (Figure 3A). The Shannon diversity index (H′) and the Pielou species evenness (J′) (Figure 3B) were also similar throughout the tidal cycle. However, the relative abundance of other taxa, mainly *Prochlorococcus*, increased at high tide (Figure 3A).

**Figure 4:**
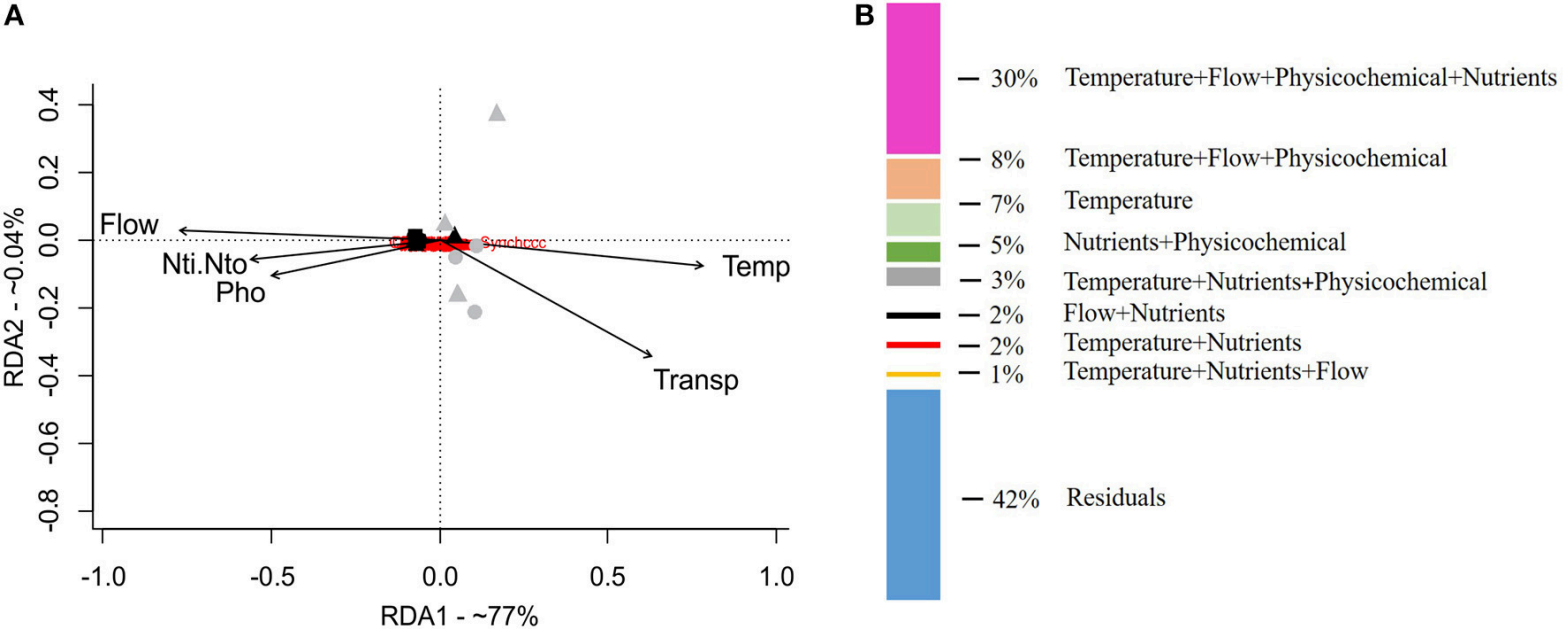
Multivariate analysis of redundancy (RDA) of the composition and abundance of cyanobacteria reads **(A)** and variation-partitioning analysis **(B)**, to quantify the relative contributions (58%) of the abiotic variables to the explanation of the composition and abundance of cyanobacteria. Black symbols, rainy season (October 2014); gray symbols, dry season (January 2015); Triangle, SER; circle, CEN; rectangle, MAR.

The redundancy analysis (RDA, Figure 4A) separated the rainy- and dry-season samples on axis 1 (77% explicability). The environmental variables that most reflected this separation between the dry and rainy seasons were freshwater inflow (Flow) and the concentrations of dissolved nutrients (NO_3_, NO_2_, PO_4_). Dry-season samples were more related to higher water temperature (Temp) and transparency (Transp). Variance partition indicated that the abiotic variables explained 58% of the variation in the community. The fraction shared by the four sets of variables (flow + temperature + nutrients + physical and chemical variables) explained around 30% of the variation. Individual environmental variables explained only small proportions of the biological data (Figure 4B).

### Functional profile

**Figure 5:**
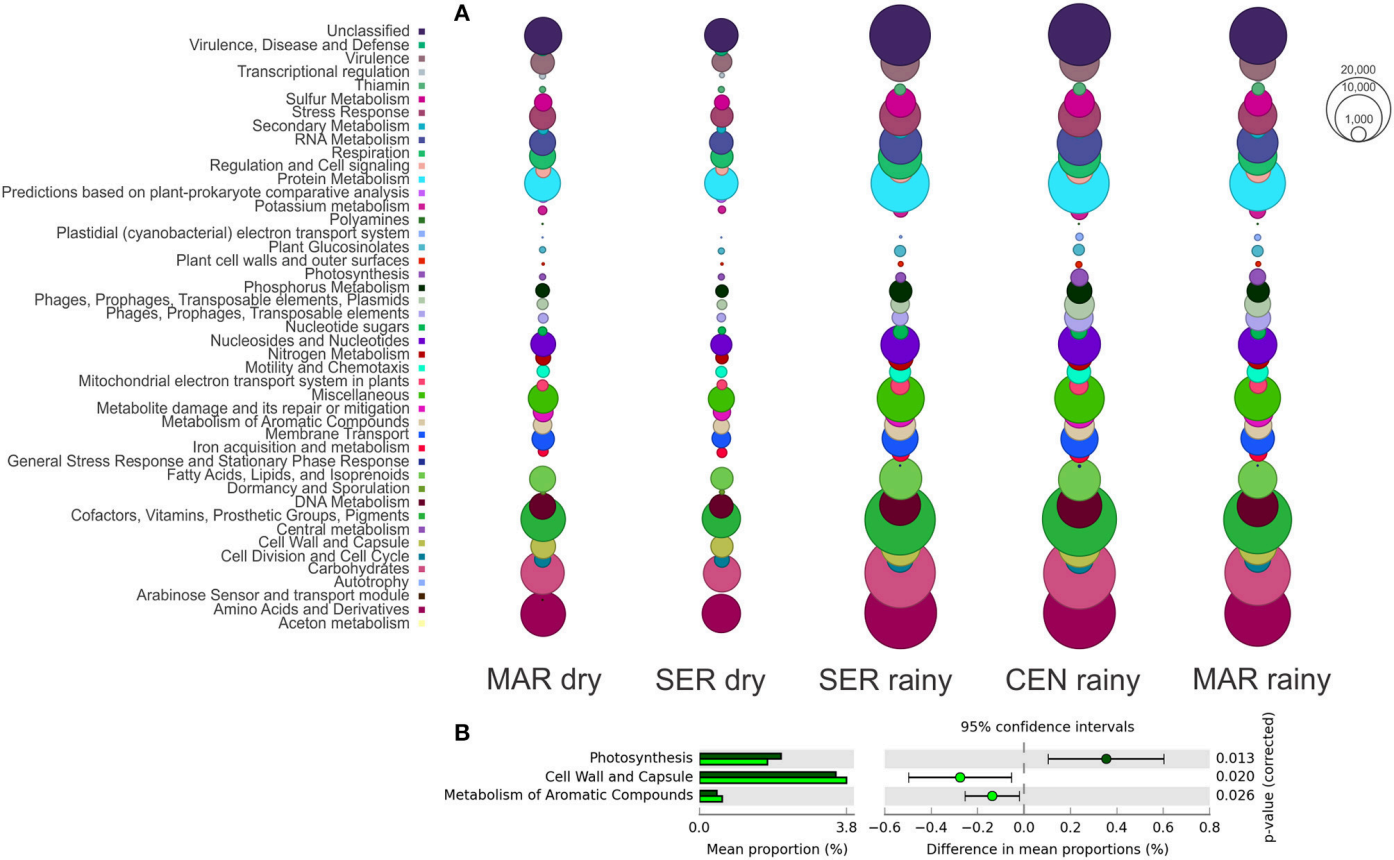
Functional profile (SEED subsystems level 1) of the cyanobacteria community **(A)** and occurrence of significant variations in relative abundances of the metabolic functions between the dry (light green bars) and rainy (dark green bars) seasons **(B)**.

**Figure 6:**
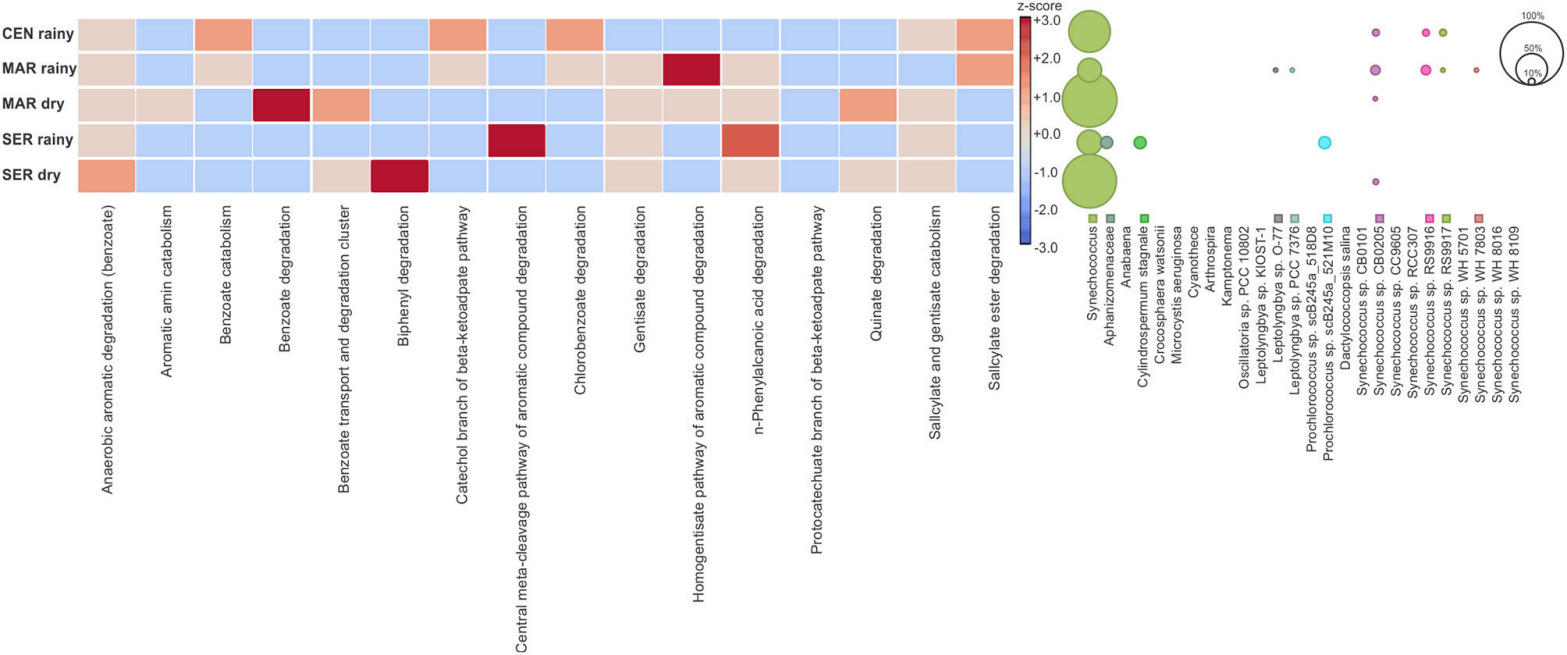
Relative abundance of cyanobacterial reads assigned to genes related to the metabolism of aromatic compounds (based on z-score transformed functional annotations). Taxonomic assignment of the reads linked to metabolism of aromatic compounds.

The SEED database was used to predict the functional profile of the cyanobacterial community in Camamu Bay. The cyanobacterial functional profile was strongly associated with the metabolism of *Synechococcus* and *Prochlorococcus*, given the predominance of these genera in the system. Sequences assigned to cofactors, vitamins, pigments, and amino acids and derivatives were abundant in both seasons (Figure 5A). The functional diversity did not vary regarding the composition of most metabolic functions. Significant differences in relative abundances between the dry and rainy seasons occurred only for photosynthesis, cell wall, and capsule, and metabolism of aromatic compounds (Figure 5B). For aromatic compounds, sequences assigned to genes involved in degradation of benzoate, chlorobenzoate, and n-phenylalkanoic acid were dominant in the samples, mainly in the dry season (Figure 6). These sequences were associated primarily with *Synechococcus*. A high representativeness of *Synechococcus* sp. CB0205 sequences was observed, especially in Central and Maraú samples (Figure 6). Only a few reads matched genes related to phosphorus metabolism (0.10%) and nitrogen metabolism (0.04%) (Figure 5A).

**Figure 7:**
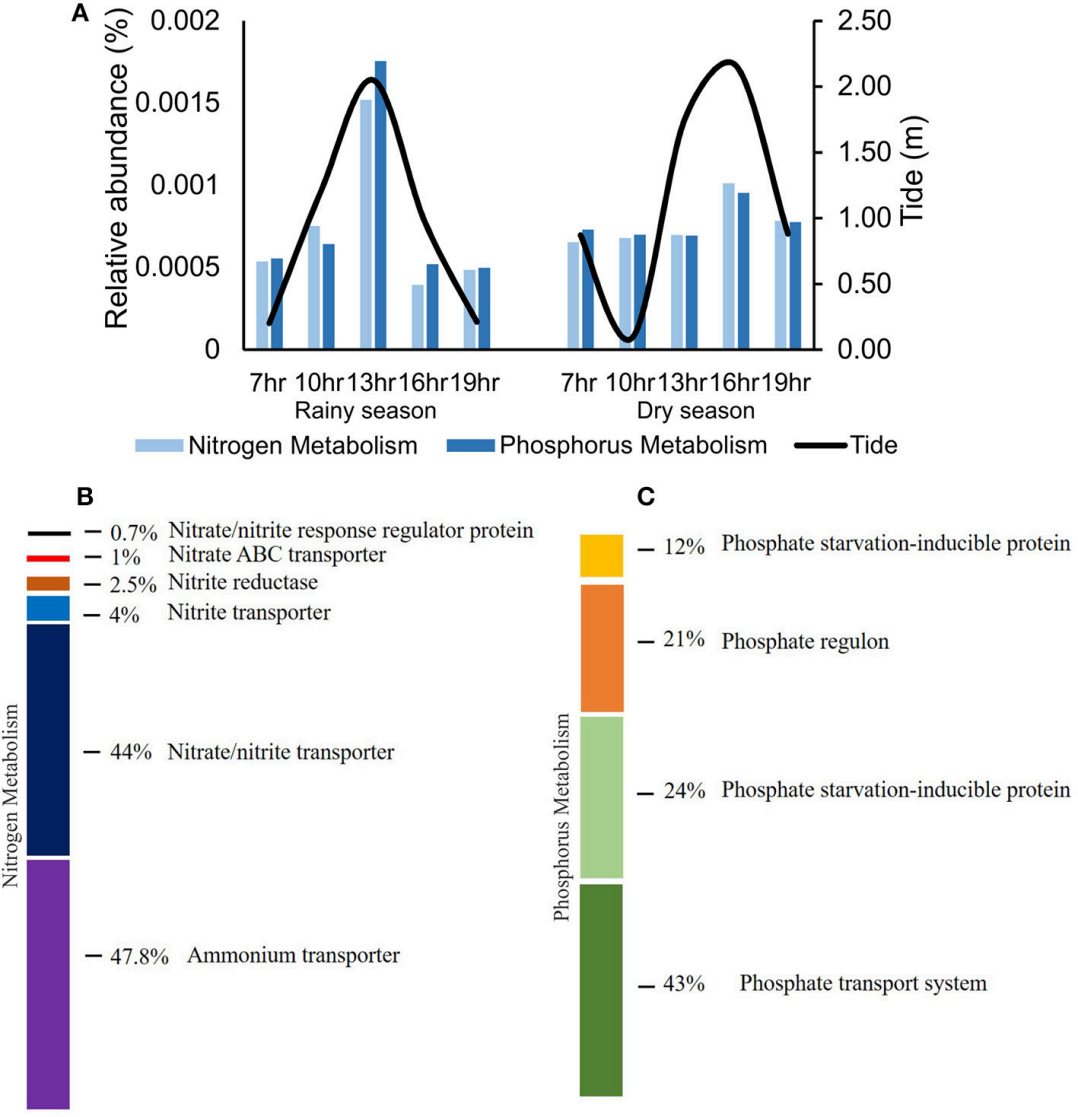
Variation of the relative abundance of cyanobacterial genes involved in nitrogen and phosphorus metabolism in tidal cycles **(A)** and composition of functions (SEED subsystems level 2) linked to nitrogen **(B)** and phosphorus **(C)** metabolism in Camamu Bay.

Comparing samples from the tidal cycles, the relative abundance of genes related to nitrogen (nitrite, nitrate, and ammonium transport) and to phosphorus metabolism increased during high tide, coinciding with the increase in *Prochlorococcus* abundance in the same samples (Figure 7A). For nitrogen metabolism, genes related to ammonium transport showed the highest relative abundance (47.8%), in dry and rainy seasons (Figure 7B). Concerning phosphorus metabolism, the predominant genes were those related to phosphate transport, in both seasons (Figure 7C).

## Discussion

Camamu Bay is a relatively pristine system (Carreira et al., 2016) with oligotrophic characteristics (Affe et al., 2018). The low nutrient concentrations in the bay are related to the influence of the tropical waters of the Brazil Current, which flows year-round along the Bahia state coast (Signorini et al., 1989; Silveira et al., 2000). The bay shows a robust marine influence because of the large mouth opening, low contribution from tributary streams throughout the year, and tidal forcing. Tidal forcing causes extensive mixing of the water column, mainly during spring tides (Amorim et al., 2011, 2015). Our data showed that although nutrient concentrations increased in the rainy season, the mean levels of total nitrogen and total phosphorus remained much lower than 11.43 and 1.61 μM, respectively, characteristic of oligotrophic estuarine environments (Silva, 2000). Thus, the environmental factors were rather homogeneous among the hydrodynamic regions and also through the tidal cycles in both seasons.

The metagenomic analysis revealed a high taxonomic diversity of cyanobacteria in the surface waters of Camamu Bay, without significant changes in the community structure (i.e., Shannon Diversity and Pielou's evenness Index), between sites, tides, or dry and rainy seasons (Figures 2B, 3B). The increase in species richness and diversity in the rainy season is linked to the increased fluvial discharges. This increased the number of freshwater taxa such as *Leptolyngbya* and *Cyanobium* (Figures 2A, 3A). We also observed a significant input of dissolved nutrients in this season (Figure 2A), transported by the rivers. These conditions are frequently associated with an increase in the support capacity of different systems (Costa et al., 2009; Majewska et al., 2017). However, picocyanobacteria predominantly described in marine habitat prevailed in the bay, and marine *Synechococcus* strains dominated the community across all samples in both seasons (90% of the sequences).

Our results agree with previous studies in other oligotrophic marine waters and estuaries worldwide. The picoplankton fraction is dominated by the unicellular coccoid *Prochlorococcus* and *Synechococcus* lineages (Platt et al., 1983; Fogg, 1995; Partensky et al., 1999; Garcia-Pichel et al., 2003; Cerino et al., 2012; Flombaum et al., 2013; Belevich et al., 2015; Díez et al., 2016), which are responsible for more than 50% of the biomass and primary productivity in coastal and estuarine environments (Johnson and Sieburth, 1979; Ray et al., 1989; Murrell and Lores, 2004). The ecological success of *Prochlorococcus* in these systems is due to its photosynthetic apparatus, which allows the species to develop in a wide range of light intensities. Similarly, specific adaptations of *Synechococcus* enable its members to develop in horizontal gradients of nutrients (Allewalt et al., 2006; Johnson et al., 2006).

*Synechococcus* is one of the most important genera of microorganisms in coastal systems, due mainly to its wide geographic distribution and importance for primary productivity (Flombaum et al., 2013; Dvorák et al., 2014). In general, the factors that control the abundance of this genus are still poorly understood. The wide variability of the group was investigated based on top-down factors such as grazing pressure, and bottom-up factors, mainly light, temperature variation, and availability of nutrients, especially nitrogen (Olson et al., 1990; Blanchot et al., 1992; Campbell and Vaulot, 1993; Scanlan and West, 2002; Dufresne et al., 2005, 2008; Paerl et al., 2011). According to Dvorák et al. (2014), the rapid generation time, and the small size and the shape of the cells allow it to compete effectively for nutrients and aid in capturing light. These factors contribute to the dominance of *Synechococcus* in surface water layers, in a wide variety of systems, under different environmental conditions year-round (Feingersch et al., 2010; Cerino et al., 2012; Mella-Flores et al., 2012; Quero and Luna, 2014).

In Camamu Bay, the dominance of *Synechococcus* may be linked to the fact that nitrate comprised ~65% of the total nitrogen in the system. It has been reported that these organisms can proliferate at low phosphate concentrations (Lomas et al., 2010; Kretz et al., 2015), and predominate in coastal systems where nitrate is the primary form of available nitrogen (Herrero et al., 2001; Wawrik et al., 2009).

Besides a high relative abundance, our data showed a high diversity of *Synechococcus*, with a total of 34 oligotypes including freshwater, euryhaline, and marine strains. Twenty-one of these strains belong to nine different clades (I-IV, VI-X, CB5, UCA, WPCI, Minus11), which are common in warm oligotrophic waters (Toledo et al., 1999; Palenik, 2001; Toledo and Palenik, 2003; Chen et al., 2004; Dufresne et al., 2008; Zwirglmaier et al., 2008; Díez et al., 2016). The dominance of euryhaline strains belonging to subclades 5.1 (clades II, IX), 5.2 (clade CB5), and 5.3 (clade X) in Camamu Bay agrees with previous studies in estuaries with different salinity gradients (Xia et al., 2015, 2017a; Mackey et al., 2017). Differences in niches among these three subclades were found in the Baltic Sea, with subclade 5.1 predominating in the marine zone, while subclades 5.2 and 5.3 were more abundant in the zones with the highest freshwater influence and dominated the low salinity-brackish transition zones, respectively (Larsson et al., 2014; Celepli et al., 2017). In this study, reads that matched *Synechococcus* sp. CB 0205 (clade CB5, subclade 5.2) were dominant in all the samples. This strain was isolated from the Chesapeake Bay in summer (Chen et al., 2006; Cai et al., 2010). It has been reported for temperate estuarine and coastal waters, and is also prevalent in polar/subpolar waters (Huang et al., 2012; Boeuf et al., 2014).

Other *Synechococcus* taxa identified in the bay include *Synechococcus* sp. WH 8109 and *Synechococcus* sp. CC 9605 (clade II, subclade 5.1), *Synechococcus* sp. RCC 307 (clade X, subclade 5.3), and *Synechococcus* sp. CC 9916 (clade XI, subclade 5.1). They are typically found in tropical or subtropical regions, in oceanic waters as well as the coastal zone in both surface and deep waters (Zwirglmaier et al., 2008; Scanlan et al., 2009; Mella-Flores et al., 2012; Xia et al., 2017b). Despite the inherent limitation of the technique for identification at low taxonomic levels, the results obtained here agree with others from similar environments. Taken together, these results suggest that the *Synechococcus* strains in Camamu Bay belong to different clades.

*Prochlorococcus* showed the highest diversity (58 strains) among the picocyanobacteria in the bay, although the genus comprised only 4% of the total sequences. The majority of the sequences belong to the clades with adaptations to intense light (HL II), in agreement with the literature for tropical and subtropical marine surface waters (e.g., Rocap et al., 2002; Bouman et al., 2006; Zwirglmaier et al., 2008; Biller et al., 2014; Babić et al., 2017). The increase in *Prochlorococcus* sequences during high tides suggests that cells are transported from ocean waters to the interior of the bay in these periods (Figure 3A). This indicates the significant influence of the marine contribution on the composition and dynamics of species in the bay.

A small group of freshwater, brackish and marine taxa comprised the remaining cyanobacteria taxa observed. Although they were responsible for the high richness of the community, these cyanobacteria occurred in low relative abundances (<5% of the sequences) (Figures 2A, 3A). High-throughput methods allowed us to assess these rare taxa, which usually are neglected in most studies using conventional techniques. These rare organisms function as a seed bank in the environment (Pedrós-Alió, 2012; Shade and Gilbert, 2015), and they can increase in abundance when environmental conditions shift and became favorable to their growth. This is the basis of the cyanobacteria bloom formation, when a few cells can rapidly proliferate and dominate the environment. For instance, *Microcystis* is well known for its capacity to form blooms in eutrophic waters, and problems with blooms of toxic strains have been reported in many estuaries throughout the world (Robson and Hamilton, 2004; Lehman et al., 2005; Otten et al., 2017; Kurobe et al., 2018). *Microcystis* was detected in Camamu Bay, mainly at the site close to the Maraú River (Figure 2A), indicating the potential for a bloom. Approximately 90 families live in the vicinity of the sampling points. Untreated domestic sewage is often discharged into the adjacent mangroves, due to the lack of basic sanitation (Pacheco, 2006). This practice can elevate the concentrations of nutrients in the system and cause a bloom formation, with direct and indirect effects on the estuarine system.

The functional contribution of the cyanobacteria in Camamu Bay was estimated using the SEED database. SEED is a subsystem approach that groups genes based on their functional roles in specific biological processes (for more information see Overbeek et al., 2005). The continuous curation of the SEED collection performed by genome-annotation specialists provides increased confidence in automatic predictions.

Considering the high degree of dominance of *Synechococcus* in Camamu Bay, only a few variations could be observed regarding the functional metagenomic analyses in both the spatial and temporal samples, among the three hydrodynamic regions and through the tidal cycles, respectively (Figure 5A). The variation in relative abundance of genes affecting nutrient metabolism (i.e., phosphorus and nitrogen) during tidal periods reflected the increase in the relative abundance of *Prochlorococcus* during high tides (Figures 3A, 6A). The relatively stable composition of cyanobacteria functional genes in the Camamu Bay, was due to the limited variation in taxonomic composition of the cyanobacterial community between the dry and rainy seasons.

The increase in the relative abundance of reads assigned to the metabolism of aromatic compounds (Figure 5B) is notable, considering that cyanobacteria are well known for their ability to degrade these compounds. Marine cyanobacteria taxa in association with aerobic organotrophic bacteria are being reported as promising in bioremediation studies, due to their ability to degrade oil (Raghukumar et al., 2001; Abed et al., 2009). This may be particularly important in Camamu Bay, considering the oil and natural-gas exploration and extraction activities on the adjacent platform, which pose a potential risk to the system in case of an oil spill in the ocean (Amorim et al., 2011).

Camamu Bay is a homogeneous system over spatial scale (hydrodynamic regions). The typical ocean-water characteristics, which influence the bay much more than the river discharges, illustrate the oligotrophic conditions of the system and the predominance of marine species in both the dry and rainy seasons. However, the greater fluvial contribution in the rainy season increased the species richness with the input of freshwater strains. These environmental characteristics provide niche conditions for a wide variety of cyanobacteria, composed of freshwater, euryhaline, and marine strains. The data acquired in this first metagenomic study revealed that strains of *Synechococcus* dominate in all seasons; many are putatively assigned to strain CB 0205, which is commonly reported from temperate estuarine and coastal waters as well as polar/subpolar waters. These findings confirmed the hypothesis that the changes in the community, although small, are influenced by the seasonal variation. We suggest that Camamu Bay can be used as a model system for studies of microbial ecology, because it is an estuarine system still preserved. Further studies are needed to clarify the geographical distributions of the different *Synechococcus* clades, and which factors drive their respective adaptations and ecological success in this system.

## Author contributions

HA wrote the draft version of the manuscript. HA, JR, and MM conceived the review. HA, JR, MM, and JN contributed to the concept and design of the study. All authors read and approved the final version of the article.

### Conflict of interest statement

The authors declare that the research was conducted in the absence of any commercial or financial relationships that could be construed as a potential conflict of interest. The handling Editor declared a shared affiliation, though no other collaboration, with one of the authors MM.

## Footnotes

^1^Instituto Nacional de Meteorologia, Centro de Previsão de Tempo e Estudos Climáticos – Instituto Nacional de Pesquisas Espaciais (INMET/CPETEC-INPE). Available online at: http://proclima.cptec.inpe.br/balanco_hidrico/balancohidrico.shtml
