# Supplementary material for: Metagenomic Analysis of Cyanobacteria in an Oligotrophic Tropical Estuary, South Atlantic
Source: Front Microbiol. 2018 Jun 26;9:1393. doi: 10.3389/fmicb.2018.01393 (PMC6029486; doi:10.3389/fmicb.2018.01393)
Supplement: Supplementary file 1 [file Data_Sheet_1.docx]

**SUPPLEMENTARY TABLE 1** Number of reads assigned in each taxonomic level of cyanobacteria in Camamu Bay

**SUPPLEMENTARY TABLE 2** Composition and relative abundance (percent) of reads of cyanobacteria in Camamu Bay XX = Presence XX = Absence

**SUPPLEMENTARY TABLE 3** Relative abundance (percent) of reads of genera of cyanobacteria and *Synechococcus* sp. strain CB0205, and reads mapped to *Synechococcus* sp. CB0205 genome in Camamu Bay

| **Taxonomic level** | **MAR dry** | **SER dry** | **SER rainy** | **CEN rainy** | **MAR rainy** | **Total reads** |
| --- | --- | --- | --- | --- | --- | --- |
| Phylum | 47140 | 30340 | 8833 | 34891 | 11943 | 133147 |
| Order | 42910 | 27913 | 7744 | 31784 | 10523 | 120874 |
| Family | 36933 | 24347 | 6725 | 27505 | 8960 | 104470 |
| Genus | 35043 | 23257 | 6279 | 26137 | 8379 | 99095 |
| Species | 10601 | 6425 | 2680 | 8070 | 3571 | 31347 |

| **Strains** | **MAR dry** | **SER dry** | **MAR rainy** | **SER rainy** | **CEN rainy** | **Total** |
| --- | --- | --- | --- | --- | --- | --- |
| *Acaryochloris marina* | **-** | **0.109** | **0.037** | **0.124** | **0.252** | **0.09** |
| *Acaryochloris* sp. CCMEE 5410 | **-** | **-** | **0.037** | **0.037** | **0.112** | **0.03** |
| *Aliterella atlantica* | **0.057** | **-** | **-** | **0.012** | **0.028** | **0.03** |
| *Anabaena cylindrica* | **-** | **-** | **0.037** | **0.012** | **0.056** | **0.01** |
| *Anabaena* sp. 90 | **-** | **-** | **0.075** | **0.012** | **0.084** | **0.02** |
| *Anabaena* sp. PCC 7108 | **-** | **-** | **0.075** | **-** | **0.028** | **0.01** |
| *Anabaena* sp. WA102 | **-** | **-** | **0.112** | **0.012** | **-** | **0.01** |
| *Anabaena variabilis* | **-** | **-** | **0.037** | **-** | **0.028** | **0.01** |
| *Aphanizomenon flos-aquae* | **0.019** | **-** | **0.037** | **-** | **0.056** | **0.02** |
| *Aphanocapsa montana* | **0.038** | **0.078** | **0.261** | **0.186** | **0.308** | **0.13** |
| *Arthrospira platensis* | **0.019** | **-** | **0.149** | **0.037** | **-** | **0.03** |
| *Calothrix parietina* | **-** | **-** | **0.037** | **0.025** | **0.056** | **0.02** |
| *Calothrix* sp. 336/3 | **-** | **0.047** | **-** | **0.012** | **0.028** | **0.02** |
| *Calothrix* sp. PCC 7103 | **0.038** | **-** | **0.187** | **0.050** | **0.056** | **0.05** |
| *Calothrix* sp. PCC 7507 | **-** | **-** | **-** | **-** | **0.056** | **0.01** |
| *Candidatus atelocyanobacterium thalassa* | **-** | **0.047** | **-** | **-** | **-** | **0.01** |
| *Candidatus Synechococcus spongiarum* | **0.038** | **0.078** | **0.075** | **0.050** | **0.084** | **0.06** |
| *Chamaesiphon minutus* | **-** | **-** | **0.075** | **0.012** | **0.056** | **0.02** |
| *Chlorogloeopsis fritschii* | **-** | **-** | **0.112** | **0.025** | **0.056** | **0.02** |
| *Chroococcidiopsis thermalis* | **-** | **-** | **-** | **0.012** | **-** | **0.00** |
| *Coleofasciculus chthonoplastes* | **0.028** | **0.093** | **0.224** | **0.037** | **0.196** | **0.08** |
| *Crinalium epipsammum* | **-** | **-** | **0.112** | **0.012** | **-** | **0.01** |
| *Crocosphaera watsonii* | **0.028** | **0.078** | **0.149** | **0.087** | **0.196** | **0.08** |
| *Cyanobacterium aponinum* | **-** | **0.062** | **0.149** | **0.012** | **0.084** | **0.04** |
| *Cyanobacterium* BACL30 MAG-120619-bin27 | **0.123** | **0.093** | **0.448** | **0.186** | **0.448** | **0.20** |
| *Cyanobacterium* PCC 7702 | **-** | **-** | **0.037** | **0.012** | **0.028** | **0.01** |
| *Cyanobacterium stanieri* | **-** | **-** | **0.075** | **0.012** | **0.028** | **0.01** |
| *Cyanobium gracile* | **0.226** | **0.327** | **0.373** | **0.458** | **0.448** | **0.34** |
| *Cyanobium* sp. CACIAM 14 | **0.094** | **0.062** | **0.187** | **0.173** | **0.056** | **0.11** |
| *Cyanobium* sp. PCC 7001 | **0.189** | **0.311** | **0.709** | **0.297** | **0.784** | **0.35** |
| *Cyanothece* sp. CCY0110 | **0.028** | **-** | **0.037** | **0.062** | **0.140** | **0.04** |
| *Cyanothece* sp. PCC 7424 | **-** | **-** | **0.149** | **0.037** | **0.084** | **0.03** |
| *Cyanothece* sp. PCC 7425 | **0.047** | **-** | **0.149** | **0.037** | **0.168** | **0.06** |
| *Cyanothece* sp. PCC 7822 | **0.028** | **-** | **0.112** | **0.062** | **0.140** | **0.05** |
| *Cyanothece* sp. PCC 8801 | **-** | **-** | **-** | **-** | **0.028** | **0.00** |
| *Cylindrospermopsis raciborskii* | **-** | **-** | **0.037** | **0.012** | **-** | **0.01** |
| *Cylindrospermopsis* sp. CR12 | **-** | **-** | **0.075** | **0.012** | **-** | **0.01** |
| *Cylindrospermum stagnale* | **-** | **-** | **0.075** | **0.025** | **0.028** | **0.02** |
| *Dactylococcopsis salina* | **0.057** | **0.047** | **0.149** | **0.087** | **0.056** | **0.07** |
| *Dolichospermum circinale* | **0.019** | **-** | **0.037** | **0.025** | **0.112** | **0.03** |
| filamentous cyanobacterium ESFC-1 | **-** | **-** | **0.075** | **0.025** | **0.084** | **0.02** |
| *Fischerella muscicola* | **-** | **-** | **0.037** | **0.012** | **0.056** | **0.01** |
| *Fischerella* sp. PCC 9339 | **0.019** | **-** | **0.075** | **-** | **-** | **0.01** |
| *Fischerella* sp. PCC 9605 | **-** | **-** | **0.075** | **-** | **0.084** | **0.02** |
| *Fortiea contorta* | **0.019** | **-** | **0.037** | **-** | **-** | **0.01** |
| *Geitlerinema* sp. PCC 7105 | **0.019** | **-** | **0.187** | **0.025** | **-** | **0.03** |
| *Geitlerinema* sp. PCC 7407 | **-** | **-** | **0.037** | **-** | **0.056** | **0.01** |
| *Geminocystis herdmanii* | **-** | **-** | **0.037** | **0.012** | **0.056** | **0.01** |
| *Geminocystis* sp. NIES-3708 | **-** | **-** | **0.037** | **0.062** | **0.252** | **0.05** |
| *Geminocystis* sp. NIES-3709 | **0.028** | **-** | **0.037** | **0.050** | **0.224** | **0.05** |
| *Gloeobacter kilaueensis* | **0.019** | **-** | **0.112** | **0.025** | **0.084** | **0.03** |
| *Gloeobacter violaceus* | **-** | **-** | **-** | **-** | **0.112** | **0.01** |
| *Gloeocapsa* sp. PCC 73106 | **-** | **-** | **0.112** | **0.074** | **0.112** | **0.04** |
| *Gloeocapsa* sp. PCC 7428 | **-** | **-** | **0.037** | **0.074** | **0.084** | **0.03** |
| *Halothece* sp. PCC 7418 | **-** | **-** | **0.187** | **0.025** | **0.028** | **0.03** |
| *Hapalosiphon* sp. MRB220 | **-** | **-** | **0.112** | **0.012** | **0.028** | **0.02** |
| *Hassallia byssoidea* | **0.151** | **0.202** | **0.784** | **0.322** | **0.588** | **0.31** |
| *Kamptonema formosum* | **-** | **-** | **-** | **-** | **0.028** | **0.00** |
| *Leptolyngbya boryana* | **0.019** | **0.078** | **0.112** | **0.025** | **0.056** | **0.04** |
| *Leptolyngbya* sp. Heron Island J | **0.038** | **-** | **0.187** | **0.050** | **-** | **0.04** |
| *Leptolyngbya* sp. JSC-1 | **-** | **-** | **0.224** | **0.124** | **0.028** | **0.05** |
| *Leptolyngbya* sp. KIOST-1 | **-** | **-** | **-** | **0.012** | **0.196** | **0.03** |
| *Leptolyngbya* sp. NIES-2104 | **-** | **-** | **-** | **0.012** | **-** | **0.00** |
| *Leptolyngbya* sp. O-77 | **-** | **-** | **0.112** | **0.012** | **0.196** | **0.04** |
| *Leptolyngbya* sp. PCC 6406 | **0.038** | **0.109** | **0.075** | **0.112** | **0.168** | **0.09** |
| *Leptolyngbya* sp. PCC 7375 | **0.066** | **0.062** | **0.299** | **0.099** | **0.084** | **0.10** |
| *Leptolyngbya* sp. PCC 7376 | **0.019** | **0.265** | **1.157** | **0.198** | **0.756** | **0.30** |
| *Limnoraphis robusta* | **-** | **-** | **-** | **0.012** | **0.112** | **0.02** |
| *Lyngbya aestuarii* | **-** | **-** | **-** | **0.025** | **0.028** | **0.01** |
| *Lyngbya confervoides* | **0.047** | **-** | **0.075** | **0.025** | **0.084** | **0.04** |
| *Lyngbya* sp. PCC 8106 | **-** | **-** | **0.112** | **0.025** | **0.028** | **0.02** |
| *Mastigocladopsis repens* | **-** | **-** | **0.037** | **0.012** | **-** | **0.01** |
| *Mastigocladus laminosus* | **-** | **-** | **-** | **0.012** | **0.084** | **0.01** |
| *Mastigocoleus testarum* | **-** | **0.047** | **0.075** | **0.025** | **0.224** | **0.05** |
| *Microcoleus* sp. PCC 7113 | **-** | **-** | **0.112** | **0.037** | **0.140** | **0.04** |
| *Microcoleus vaginatus* | **0.028** | **-** | **0.075** | **0.037** | **0.056** | **0.03** |
| *Microcystis aeruginosa* | **0.491** | **0.233** | **0.448** | **0.186** | **0.336** | **0.34** |
| *Microcystis* sp. T1-4 | **0.019** | **-** | **-** | **-** | **0.028** | **0.01** |
| *Moorea bouillonii* | **-** | **-** | **-** | **-** | **0.028** | **0.00** |
| *Moorea producens* | **0.028** | **0.062** | **0.187** | **0.124** | **0.196** | **0.09** |
| *Myxosarcina* sp. GI1 | **0.019** | **0.062** | **0.075** | **0.062** | **0.140** | **0.06** |
| *Neosynechococcus sphagnicola* | **-** | **-** | **0.037** | **0.012** | **0.028** | **0.01** |
| *Nodosilinea nodulosa* | **0.151** | **0.140** | **0.299** | **0.186** | **0.308** | **0.19** |
| *Nodularia spumigena* | **-** | **-** | **0.075** | **-** | **0.028** | **0.01** |
| *Nostoc commune* | **-** | **-** | **0.037** | **-** | **-** | **0.00** |
| *Nostoc punctiforme* | **-** | **-** | **-** | **0.012** | **0.028** | **0.01** |
| *Nostoc* sp. CAVN2 | **-** | **-** | **-** | **-** | **0.028** | **0.00** |
| *Nostoc* sp. NIES-3756 | **-** | **-** | **-** | **-** | **0.084** | **0.01** |
| *Nostoc* sp. PCC 7120 | **-** | **-** | **0.075** | **-** | **-** | **0.01** |
| *Nostoc* sp. PCC 7524 | **0.028** | **0.062** | **0.149** | **0.025** | **0.056** | **0.05** |
| *Nostoc* sp. *'Peltigera membranacea cyanobiont'* | **-** | **-** | **-** | **0.012** | **-** | **0.00** |
| *Oscillatoria acuminata* | **0.047** | **0.047** | **0.075** | **0.037** | **0.056** | **0.05** |
| *Oscillatoria nigro-viridis* | **-** | **-** | **0.037** | **0.012** | **0.028** | **0.01** |
| *Oscillatoria* sp. PCC 10802 | **0.019** | **-** | **0.149** | **0.062** | **0.112** | **0.05** |
| [*Oscillatoria*] sp. PCC 6506 | **-** | **-** | **-** | **-** | **0.028** | **0.00** |
| Oscillatoriales cyanobacterium JSC-12 | **0.038** | **-** | **0.075** | **-** | **0.056** | **0.03** |
| Oscillatoriales cyanobacterium MTP1 | **-** | **-** | **0.075** | **0.025** | **0.028** | **0.02** |
| *Phormidesmis priestleyi* | **-** | **0.047** | **0.075** | **0.025** | **0.056** | **0.03** |
| *Phormidium* sp. OSCR | **-** | **-** | **0.037** | **-** | **0.028** | **0.01** |
| *Planktothricoides* sp. SR001 | **-** | **-** | **0.187** | **0.124** | **0.084** | **0.06** |
| *Planktothrix agardhii* | **-** | **-** | **-** | **0.012** | **0.056** | **0.01** |
| *Planktothrix prolifica* | **-** | **-** | **-** | **-** | **0.028** | **0.00** |
| *Pleurocapsa minor* | **-** | **-** | **0.075** | **-** | **-** | **0.01** |
| *Pleurocapsa* sp. PCC 7319 | **0.047** | **0.078** | **0.187** | **0.025** | **0.168** | **0.07** |
| *Prochlorococcus marinus* | **1.613** | **1.837** | **2.201** | **1.648** | **2.324** | **1.80** |
| *Prochlorococcus* sp. MIT 0601 | **0.019** | **0.047** | **0.075** | **-** | **0.056** | **0.03** |
| *Prochlorococcus* sp. MIT 0604 | **-** | **-** | **0.075** | **0.012** | **-** | **0.01** |
| *Prochlorococcus* sp. MIT 0701 | **0.132** | **0.078** | **0.112** | **0.074** | **0.112** | **0.10** |
| *Prochlorococcus* sp. MIT 0702 | **-** | **-** | **0.037** | **0.012** | **0.028** | **0.01** |
| *Prochlorococcus* sp. MIT 0703 | **-** | **-** | **-** | **0.012** | **0.028** | **0.01** |
| *Prochlorococcus* sp. MIT 0801 | **0.047** | **0.078** | **0.075** | **-** | **-** | **0.04** |
| *Prochlorococcus* sp. scB241_526B19 | **-** | **-** | **-** | **-** | **0.028** | **0.00** |
| *Prochlorococcus* sp. scB241_526D20 | **-** | **-** | **0.037** | **-** | **-** | **0.00** |
| *Prochlorococcus* sp. scB241_526N9 | **-** | **-** | **-** | **0.012** | **-** | **0.00** |
| *Prochlorococcus* sp. scB241_527E14 | **-** | **-** | **0.037** | **-** | **-** | **0.00** |
| *Prochlorococcus* sp. scB241_527L15 | **0.028** | **0.047** | **0.112** | **0.037** | **-** | **0.04** |
| *Prochlorococcus* sp. scB241_527L22 | **-** | **-** | **0.037** | **-** | **0.028** | **0.01** |
| *Prochlorococcus* sp. scB241_527N11 | **-** | **-** | **0.037** | **0.025** | **-** | **0.01** |
| *Prochlorococcus* sp. scB241_528J8 | **-** | **0.047** | **0.224** | **0.012** | **0.112** | **0.04** |
| *Prochlorococcus* sp. scB241_528N17 | **-** | **-** | **0.037** | **-** | **0.028** | **0.01** |
| *Prochlorococcus* sp. scB241_528O2 | **0.019** | **0.047** | **-** | **0.025** | **0.028** | **0.03** |
| *Prochlorococcus* sp. scB241_528P14 | **-** | **-** | **-** | **-** | **0.028** | **0.00** |
| *Prochlorococcus* sp. scB241_528P18 | **-** | **-** | **0.075** | **0.012** | **0.056** | **0.02** |
| *Prochlorococcus* sp. scB241_529B19 | **-** | **-** | **-** | **-** | **0.028** | **0.00** |
| *Prochlorococcus* sp. scB241_529C4 | **-** | **-** | **-** | **0.012** | **-** | **0.00** |
| *Prochlorococcus* sp. scB241_529D18 | **-** | **-** | **0.037** | **0.012** | **-** | **0.01** |
| *Prochlorococcus* sp. scB241_529J15 | **-** | **-** | **0.037** | **-** | **0.084** | **0.01** |
| *Prochlorococcus* sp. scB241_529O19 | **-** | **-** | **-** | **0.012** | **-** | **0.00** |
| *Prochlorococcus* sp. scB243_495G23 | **-** | **-** | **0.037** | **-** | **-** | **0.00** |
| *Prochlorococcus* sp. scB243_495I8 | **0.104** | **0.202** | **0.112** | **0.074** | **0.084** | **0.11** |
| *Prochlorococcus* sp. scB243_495K23 | **-** | **-** | **0.075** | **-** | **0.056** | **0.01** |
| *Prochlorococcus* sp. scB243_495N3 | **-** | **-** | **-** | **0.012** | **-** | **0.00** |
| *Prochlorococcus* sp. scB243_495N4 | **-** | **-** | **-** | **-** | **0.028** | **0.00** |
| *Prochlorococcus* sp. scB243_495P20 | **-** | **-** | **-** | **-** | **0.028** | **0.00** |
| *Prochlorococcus* sp. scB243_496A2 | **-** | **-** | **0.075** | **-** | **-** | **0.01** |
| *Prochlorococcus* sp. scB243_496M6 | **-** | **-** | **0.037** | **-** | **-** | **0.00** |
| *Prochlorococcus* sp. scB243_497E17 | **0.028** | **0.171** | **0.410** | **0.087** | **0.140** | **0.12** |
| *Prochlorococcus* sp. scB243_497J18 | **-** | **-** | **-** | **-** | **0.056** | **0.01** |
| *Prochlorococcus* sp. scB243_498I20 | **-** | **-** | **0.075** | **-** | **0.028** | **0.01** |
| *Prochlorococcus* sp. scB243_498J20 | **0.104** | **0.109** | **0.112** | **0.074** | **0.140** | **0.10** |
| *Prochlorococcus* sp. scB243_498L10 | **-** | **-** | **0.187** | **0.012** | **0.140** | **0.04** |
| *Prochlorococcus* sp. scB243_498N4 | **-** | **-** | **-** | **0.012** | **-** | **0.00** |
| *Prochlorococcus* sp. scB243_498P3 | **-** | **-** | **0.075** | **-** | **0.028** | **0.01** |
| *Prochlorococcus* sp. scB245a_518A17 | **0.019** | **0.047** | **-** | **-** | **0.084** | **0.03** |
| *Prochlorococcus* sp. scB245a_518D8 | **0.274** | **0.949** | **4.142** | **0.768** | **3.696** | **1.26** |
| *Prochlorococcus* sp. scB245a_518I6 | **-** | **-** | **0.037** | **-** | **-** | **0.00** |
| *Prochlorococcus* sp. scB245a_518J7 | **-** | **-** | **0.037** | **-** | **0.028** | **0.01** |
| *Prochlorococcus* sp. scB245a_518K17 | **-** | **-** | **0.037** | **-** | **-** | **0.00** |
| *Prochlorococcus* sp. scB245a_518O7 | **0.057** | **-** | **0.075** | **0.074** | **0.140** | **0.06** |
| *Prochlorococcus* sp. scB245a_519A13 | **-** | **-** | **0.037** | **0.012** | **-** | **0.01** |
| *Prochlorococcus* sp. scB245a_519E23 | **0.028** | **-** | **-** | **0.050** | **0.028** | **0.03** |
| *Prochlorococcus* sp. scB245a_519G16 | **-** | **-** | **0.075** | **-** | **-** | **0.01** |
| *Prochlorococcus* sp. scB245a_519L21 | **0.038** | **-** | **0.037** | **-** | **-** | **0.02** |
| *Prochlorococcus* sp. scB245a_519O11 | **-** | **-** | **0.037** | **-** | **0.084** | **0.01** |
| *Prochlorococcus* sp. scB245a_519O21 | **-** | **-** | **-** | **-** | **0.028** | **0.00** |
| *Prochlorococcus* sp. scB245a_520F22 | **-** | **-** | **0.037** | **-** | **-** | **0.00** |
| *Prochlorococcus* sp. scB245a_521B10 | **0.198** | **0.374** | **0.261** | **0.173** | **0.056** | **0.22** |
| *Prochlorococcus* sp. scB245a_521C8 | **-** | **-** | **0.037** | **-** | **-** | **0.00** |
| *Prochlorococcus* sp. scB245a_521K15 | **0.028** | **-** | **-** | **-** | **-** | **0.01** |
| *Prochlorococcus* sp. scB245a_521M10 | **-** | **-** | **0.037** | **0.025** | **0.140** | **0.03** |
| *Prochlorococcus* sp. W2 | **0.019** | **-** | **0.037** | **-** | **0.028** | **0.01** |
| *Prochlorococcus* sp. W8 | **-** | **-** | **0.037** | **0.037** | **-** | **0.01** |
| *Pseudanabaena biceps* | **-** | **-** | **0.037** | **-** | **-** | **0.00** |
| *Pseudanabaena* sp. PCC 6802 | **0.019** | **0.062** | **0.112** | **0.050** | **0.056** | **0.05** |
| *Pseudanabaena* sp. PCC 7367 | **0.038** | **0.062** | **0.075** | **0.025** | **0.056** | **0.04** |
| *Pseudanabaena* sp. 'Roaring Creek' | **-** | **-** | **0.037** | **0.012** | **-** | **0.01** |
| *Raphidiopsis brookii* | **-** | **-** | **0.037** | **-** | **0.056** | **0.01** |
| *Rivularia* sp. PCC 7116 | **-** | **-** | **-** | **0.050** | **0.112** | **0.03** |
| *Rubidibacter lacunae* | **0.028** | **-** | **0.075** | **0.012** | **0.056** | **0.03** |
| *Scytonema millei* | **0.085** | **-** | **0.261** | **0.099** | **0.420** | **0.12** |
| *Scytonema tolypothrichoides* | **-** | **-** | **-** | **-** | **0.028** | **0.00** |
| [*Scytonema hofmanni*] UTEX B 1581 | **0.019** | **-** | **0.075** | **0.062** | **0.028** | **0.03** |
| *Spirulina subsalsa* | **-** | **0.047** | **-** | **-** | **0.056** | **0.02** |
| *Stanieria cyanosphaera* | **-** | **0.047** | **0.075** | **0.012** | **0.028** | **0.02** |
| *Synechococcus* sp. BL107 | **0.547** | **0.591** | **0.261** | **0.496** | **0.196** | **0.48** |
| *Synechococcus* sp. CB0101 | **1.472** | **1.245** | **1.119** | **1.450** | **1.400** | **1.38** |
| *Synechococcus* sp. CB0205 | **46.477** | **36.576** | **38.657** | **40.310** | **40.885** | **41.55** |
| *Synechococcus* sp. CC9311 | **0.160** | **0.374** | **0.187** | **0.335** | **0.252** | **0.26** |
| *Synechococcus* sp. CC9605 | **6.716** | **7.626** | **3.396** | **6.406** | **2.828** | **6.10** |
| *Synechococcus* sp. CC9616 | **0.292** | **0.358** | **0.187** | **0.520** | **0.196** | **0.34** |
| *Synechococcus* sp. CC9902 | **0.330** | **0.436** | **0.299** | **0.458** | **0.168** | **0.36** |
| *Synechococcus* sp. GFB01 | **0.104** | **0.187** | **0.336** | **0.173** | **0.420** | **0.19** |
| *Synechococcus* sp. JA-2-3B'a(2-13) | **0.028** | **-** | **-** | **-** | **-** | **0.01** |
| *Synechococcus* sp. JA-3-3Ab | **-** | **-** | **-** | **-** | **0.028** | **0.00** |
| *Synechococcus* sp. KORDI-100 | **0.566** | **0.514** | **0.410** | **0.595** | **0.532** | **0.55** |
| *Synechococcus* sp. KORDI-49 | **0.717** | **0.872** | **0.485** | **0.954** | **0.896** | **0.81** |
| *Synechococcus* sp. KORDI-52 | **3.971** | **4.405** | **2.127** | **3.631** | **2.072** | **3.60** |
| *Synechococcus* sp. Minos11 | **-** | **-** | **-** | **-** | **0.028** | **0.00** |
| *Synechococcus* sp. NKBG042902 | **-** | **-** | **-** | **-** | **0.028** | **0.00** |
| *Synechococcus* sp. NKBG15041c | **-** | **-** | **0.037** | **-** | **0.028** | **0.01** |
| *Synechococcus* sp. PCC 6312 | **-** | **-** | **0.112** | **-** | **0.112** | **0.02** |
| *Synechococcus* sp. PCC 7002 | **-** | **-** | **-** | **-** | **0.056** | **0.01** |
| *Synechococcus* sp. PCC 7335 | **-** | **0.109** | **0.037** | **0.037** | **0.028** | **0.04** |
| *Synechococcus* sp. PCC 7336 | **0.066** | **0.078** | **0.149** | **0.136** | **0.168** | **0.11** |
| *Synechococcus* sp. PCC 7502 | **-** | **-** | **0.075** | **0.012** | **0.028** | **0.01** |
| *Synechococcus* sp. RCC307 | **6.782** | **11.767** | **5.933** | **9.281** | **5.293** | **8.20** |
| *Synechococcus* sp. RS9916 | **8.848** | **6.459** | **10.037** | **9.504** | **11.117** | **8.89** |
| *Synechococcus* sp. RS9917 | **1.094** | **1.463** | **2.052** | **1.698** | **2.072** | **1.52** |
| *Synechococcus* sp. RS9920 | **-** | **-** | **0.037** | **-** | **-** | **0.00** |
| *Synechococcus* sp. UTEX 2973 | **-** | **-** | **0.037** | **-** | **-** | **0.00** |
| *Synechococcus* sp. WH 5701 | **0.151** | **0.311** | **0.224** | **0.335** | **0.252** | **0.25** |
| *Synechococcus* sp. WH 7803 | **0.755** | **0.545** | **0.933** | **0.892** | **0.644** | **0.75** |
| *Synechococcus* sp. WH 7805 | **1.849** | **1.712** | **1.828** | **2.107** | **1.792** | **1.88** |
| *Synechococcus* sp. WH 8016 | **0.755** | **0.840** | **0.634** | **0.892** | **0.756** | **0.80** |
| *Synechococcus* sp. WH 8020 | **0.528** | **0.296** | **0.410** | **0.397** | **0.616** | **0.45** |
| *Synechococcus* sp. WH 8102 | **0.472** | **0.638** | **0.373** | **0.558** | **0.420** | **0.51** |
| *Synechococcus* sp. WH 8103 | **0.462** | **0.265** | **0.261** | **0.434** | **0.196** | **0.37** |
| *Synechococcus* sp. WH 8109 | **10.707** | **13.619** | **4.515** | **8.947** | **4.397** | **9.60** |
| *Synechocystis* sp. PCC 6714 | **-** | **0.062** | **0.075** | **0.062** | **0.056** | **0.04** |
| *Synechocystis* sp. PCC 6803 | **0.019** | **-** | **0.075** | **-** | **-** | **0.01** |
| *Synechocystis* sp. PCC 7509 | **0.028** | **-** | **-** | **-** | **-** | **0.01** |
| *Thermosynechococcus elongatus* | **-** | **-** | **-** | **0.012** | **0.028** | **0.01** |
| *Thermosynechococcus* sp. NK55a | **-** | **-** | **-** | **0.012** | **-** | **0.00** |
| *Tolypothrix bouteillei* | **-** | **-** | **0.075** | **0.025** | **0.056** | **0.02** |
| *Tolypothrix campylonemoides* | **-** | **0.047** | **0.075** | **0.025** | **-** | **0.02** |
| *Tolypothrix* sp. PCC 7601 | **-** | **-** | **0.149** | **0.025** | **0.112** | **0.03** |
| *Trichodesmium erythraeum* | **0.613** | **1.354** | **2.388** | **0.768** | **2.212** | **1.14** |
| uncultured marine type-A *Synechococcus* GOM 3O12 | **0.028** | **-** | **0.037** | **-** | **0.056** | **0.02** |
| *Xenococcus* sp. PCC 7305 | **0.047** | **0.078** | **0.187** | **0.074** | **0.168** | **0.09** |

| **Genus** | **MAR dry** | **SER dry** | **SER rainy** | **CEN rainy** | **MAR rainy** | **Total reads** |
| --- | --- | --- | --- | --- | --- | --- |
| *Microcystis* | 0.17 | 0.05 | 0.05 | 0.04 | 0.04 | 0.30 |
| *Trichodesmium* | 0.21 | 0.28 | 0.20 | 0.20 | 0.20 | 0.80 |
| *Leptolyngbya* | 0.06 | 0.11 | 0.17 | 0.19 | 0.19 | 0.60 |
| *Cyanobium* | 0.17 | 0.14 | 0.24 | 0.11 | 0.11 | 0.70 |
| Others | 0.52 | 0.37 | 0.91 | 0.77 | 0.77 | 2.00 |
| *Prochlorococcus* | 0.93 | 0.83 | 0.86 | 0.91 | 0.80 | 5.12 |
| *Synechococcus* | 31.75 | 18.72 | 23.33 | 8.88 | 6.44 | 90.48 |
| *Synechococcus* sp. CB0205 | 10.45 | 7.75 | 11.73 | 9.32 | 12.22 | 41.17 |
| *Synechococcus* sp. CB0205 genome | 10.08 | 8.37 | 9.2 | 7.93 | 10.76 | - |
